# LabVIEW-based sequential-injection analysis system for the determination of trace metals by square-wave anodic and adsorptive stripping voltammetry on mercury-film electrodes

**DOI:** 10.1155/S1463924603000233

**Published:** 2003

**Authors:** Anastasios Economou, Anastasios Voulgaropoulos

**Affiliations:** Laboratory of Analytical Chemistry Department of Chemistry Aristotle University of Thessaloniki Thessaloni GR-541 24 Greece

## Abstract

The development of a dedicated automated sequential-injection analysis apparatus for anodic stripping voltammetry (ASV) and adsorptive stripping voltammetry (AdSV) is reported. The instrument comprised a peristaltic pump, a multiposition selector valve and a home-made potentiostat and used a mercury-film electrode as the working electrodes in a thin-layer electrochemical detector. Programming of the experimental sequence was performed in LabVIEW 5.1. The sequence of operations included formation of the mercury film, electrolytic or adsorptive accumulation of the analyte on the electrode surface, recording of the voltammetric current-potential response, and cleaning of the electrode. The stripping step was carried out by applying a square-wave (SW) potential-time excitation signal to the working electrode. The instrument allowed unattended operation since multiple-step sequences could be readily implemented through the purpose-built software. The utility of the analyser was tested for the determination of copper(II), cadmium(II), lead(II) and zinc(II) by SWASV and of nickel(II), cobalt(II) and uranium(VI) by SWAdSV.


Journal of Automated Methods & Management in Chemistry
Vol. 25, No. 6 (November–December 2003) pp. 133–140

Taylor & Francis

Taylor & Francis Group

### LabVIEW-based sequential-injection analysis system for the determination of trace metals by square-wave anodic and adsorptive stripping voltammetry on mercury-film electrodes

**Anastasios Economou and Anastasios Voulgaropoulos**

*Laboratory of Analytical Chemistry, Department of Chemistry, Aristotle University of Thessaloniki, GR-541 24 Thessaloni, Greece*

*The development of a dedicated automated sequential-injection analysis apparatus for anodic stripping voltammetry (ASV) and adsorptive stripping voltammetry (AdSV) is reported. The instrument comprised a peristaltic pump, a multiposition selector valve and a home-made potentiostat and used a mercury-film electrode as the working electrodes in a thin-layer electrochemical detector. Programming of the experimental sequence was performed in LabVIEW 5.1. The sequence of operations included formation of the mercury film, electrolytic or adsorptive accumulation of the analyte on the electrode surface, recording of the voltammetric current–potential response, and cleaning of the electrode. The stripping step was carried out by applying a square-wave (SW) potential–time excitation signal to the working electrode. The instrument allowed unattended operation since multiple-step sequences could be readily implemented through the purpose-built software. The utility of the analyser was tested for the determination of copper(II), cadmium(II), lead(II) and zinc(II) by SWASV and of nickel(II), cobalt(II) and uranium(VI) by SWAdSV.*

### Introduction

Sequential-injection analysis (SIA) is the most recent version in the field of flow analysis techniques [1]. It is based on the sequential aspiration of solutions in a holding coil as separate zones, followed by the transport of the zones towards the detector through a reaction coil. The advantages of SIA over conventional flow-injection analysis (FIA) are the reduced consumption of reagents, the convenience in varying the chemical and instrumental operating parameters, the ease of calibration, and the versatility and simplicity afforded by the single-channel manifold. The SIA principle is applicable to different detection schemes, such as ultraviolet light-vis spectrophotometry, chemiluminescence, infrared spectrometry, atomic absorption and potentiometry [1]. In addition, it has been demonstrated that the SIA methodology is ideally suited to electrochemical stripping techniques that employ a preconcentration stage of the analyte on the working electrode before the actual measurement [2,3]. However, a major requirement of SIA is that computer control of the whole experimental sequence is imperative to achieve reproducible sample aspiration and delivery.

Anodic stripping voltammetry (ASV) on mercury-film electrodes (MFEs) has been recognized as a powerful technique for the determination of trace metals [4]. Moreover, over the last two decades, MFEs have found applications in the field of adsorptive stripping voltammetry (AdSV) [5]. In general, MFEs have been shown to possess unique advantages as detectors in flow-through electro-analysis when compared with the traditional hanging mercury drop electrode (HMDE) such as mechanical stability, ruggedness, simplicity of construction, low cost, scope for different cell designs and convenient cleaning of the surface. When combined with the square-wave (SW) mode of potential scanning, their main drawback of relatively high background currents is also addressed. The SW modulation additionally offers high speed, enhanced insensitivity to dissolved oxygen and higher analytical sensitivity [6].

However, more widespread use of MFEs in AdSV is limited by the complexity and time overhead in plating, cleaning and stripping of the mercury film. This drawback can be conveniently addressed by computer-controlled flow systems in which the different steps of the analysis are automated [7–10]. The present work reports the development of a fully automated SIA instrument for square-wave adsorptive stripping voltammetry (SWAdSV) on MFEs. The instrument made use of a home-made potentiostat, a peristaltic pump and a multiport selection valve. The formation of the mercury film, the preconcentration of the analytical species on the electrode, the presentation of the results and the reactivation of the electrode were performed by software developed in-house in LabVIEW 5.1. The LabVIEW software package is ideally suited to the task of automating analytical instrumentation [11–14]. The user friendliness of the user interface (front panel of the programme) offered excellent flexibility and simplified the selection of the operational parameters, the data acquisition, and the presentation and evaluation of the results.

*Journal of Automated Methods & Management in Chemistry* ISSN 1463–9246 print/ISSN 1464–5068 online © 2004 Taylor & Francis Ltd
http://www.tandf.co.uk/journals
DOI: 10.1080/14639240410001699173

### Experimental

*Reagents*

All the reagents were of analytical grade and deionized water was used throughout the experiments. Stock $1000\,mg\,l^{-1}$ atomic absorption standard metal ion solutions were used for the determination of cadmium (Cd) (II), lead (Pb) (II), copper (Cu) (II), zinc (Zn) (II), nickel (Ni) (II) and cobalt (Co) (II). Uranyl nitrate (dissolved in a minimum amount of concentrated $HNO_3$) was used for the preparation of a $1000\,mg\,l^{-1}$ uranium (U) (VI) solution. The acetate buffer ($0.1\,mol\,l^{-1}$ in total acetate species, pH 4.5) was prepared by mixing the appropriate amounts of concentrated $CH_3COOH$ and $NH_3$. The acetate in both ASV and AdSV was used without any deoxygenation. The ammonia buffer ($0.1\,mol\,l^{-1}$ in total ammonium species, pH 9.2) was prepared by mixing the appropriate amounts of concentrated $NH_3$ and HCl; this buffer was deoxygenated by purging the solution with nitrogen. Stock $0.1\,mol\,l^{-1}$ solutions of dimethylglyoxime and cupferron were prepared by dissolving the appropriate amount of the solids in absolute ethanol and water, respectively. The stock $1000\,mg\,l^{-1}$ mercury (Hg) (II) plating solution was prepared from $HgCl_2{\cdot}6H_2O$ and the working $200\,mg\,l^{-1}$ and $10\,mg\,l^{-1}$ Hg(II) solutions were prepared in $1\,mol\,l^{-1}$ HCl.

*Instrumentation*

A schematic diagram of the complete SIA instrument is shown in figure 1(a). Solution aspiration and delivery were accomplished by means of a peristaltic pump (Gilson Minipuls 3, Villiers le Bel, France). A 10-port valve (Vici-Valco, Schenkon, Switzerland) served as a selection valve. The electrochemical thin-layer flow cell was designed and constructed in-house (figure 1b). The working electrode was a glassy carbon disk (2 mm in diameter), the reference electrode was a gel-based Ag/AgCl and the counter electrode was a stainless steel tube also serving as the solution outlet. The thin layer flow channel was defined by a 0.5-mm Teflon spacer placed between the two parts forming the cell. The electrodes were connected to an adder-type home-made potentiostat with provision for external potential input and current output connections, similar to the

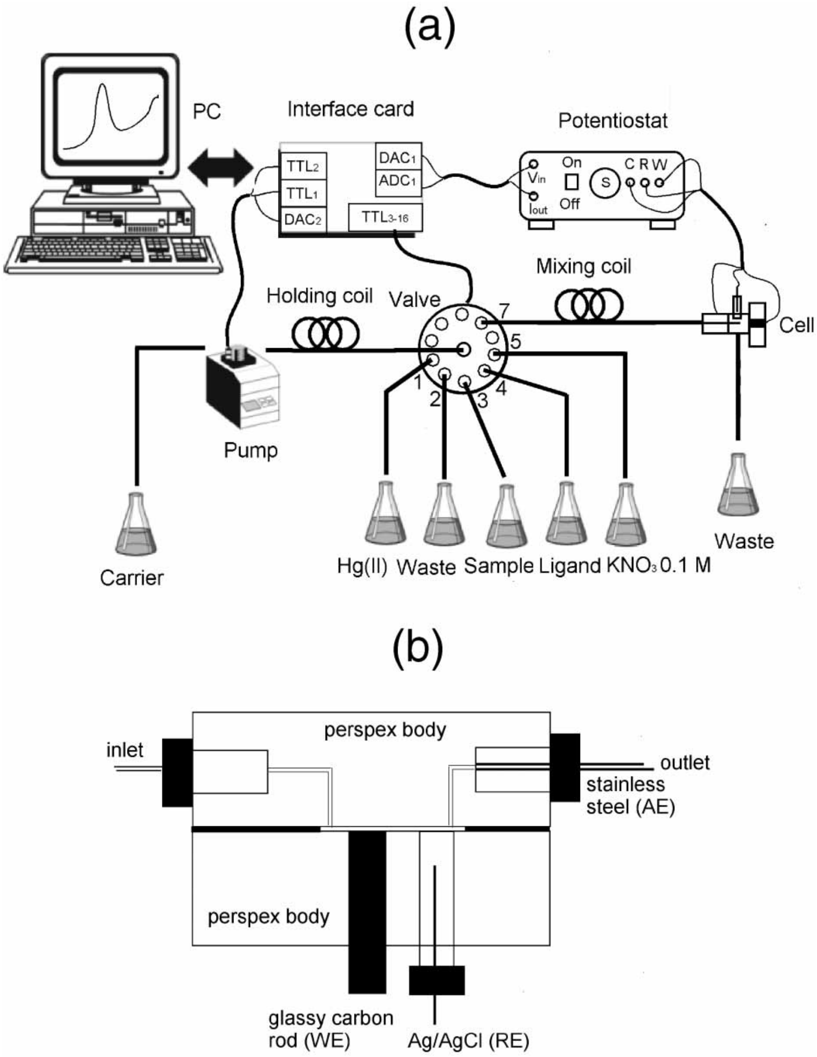

*Figure 1. (a) Experimental configuration of the SIA apparatus for stripping voltammetry; (b) schematic diagram of the thin-layer electrochemical flow-cell.*

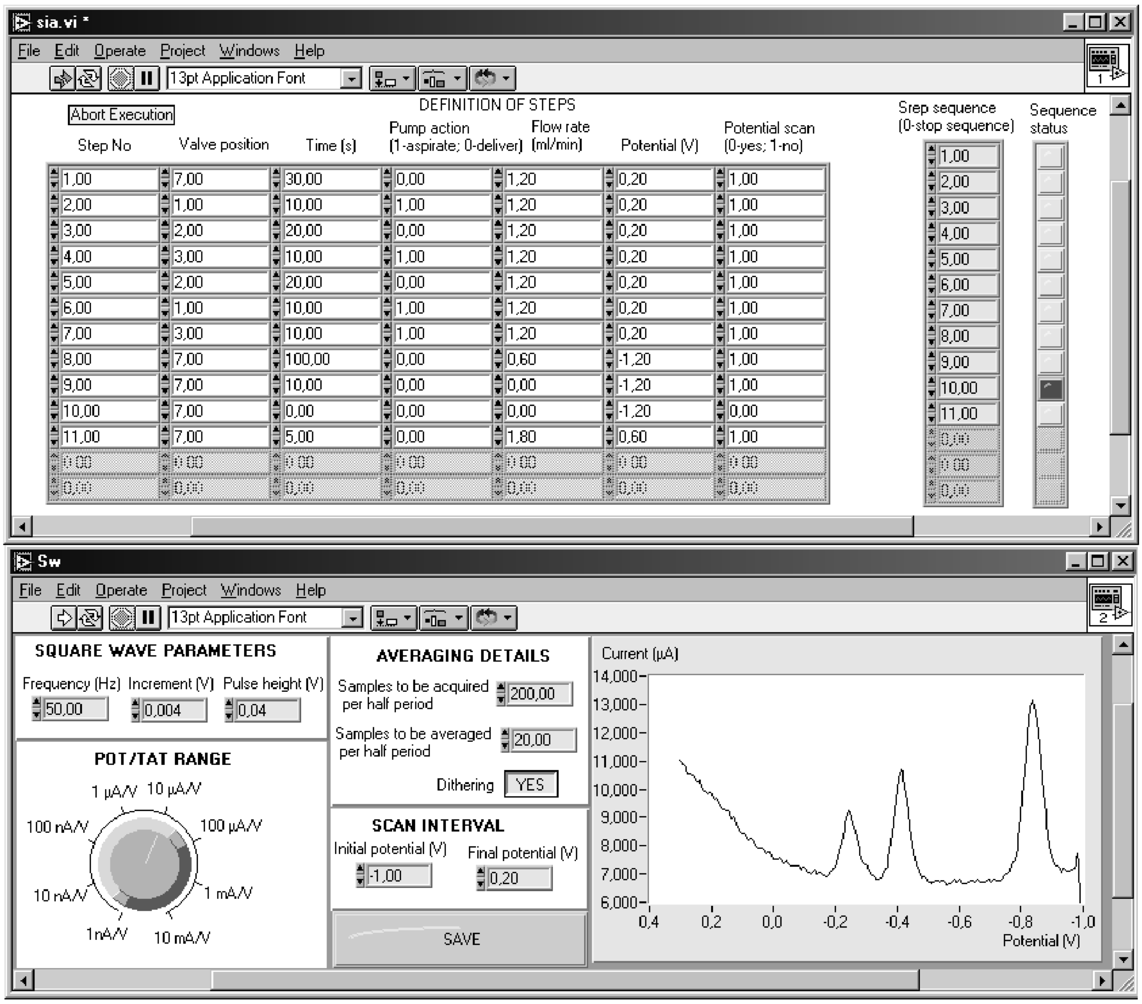

*Figure 2. Front panel (user interface) of the control and acquisition programme developed.*

one reported earlier [15]. The pump, valve and potentiostat were interfaced to a Pentium computer through a multifunction interface card (6025 E PCI, National Instruments, Austin, TX, USA). The pump made use of two TTL signals (one for on/off and another for the forward/reverse direction) and one DAC channel (for speed control). The speed of the pump was calibrated in ml $min^{-1}$. The valve was controlled by a BCD code that allowed selection of the port and reading back the port selected. This operation required 14 TTL lines. The potentiostat necessitated the use of one DAC channel (for potential control) and an ADC channel (for current acquisition).

*Software*

Programming was accomplished in LabVIEW 5.1 (National Instruments). The computer screen displayed two so-called ‘front panels’ (figure 2). The top front panel, called sia.vi, is the front panel of the main control programme of the analyser. The bottom front panel, called sw, is the front panel of the programme that performed the actual voltammetric measurement.

In SIA, one must first define a number of *steps*. In the programme sia.vi, a step was an array of commands for the pump (speed, direction, time of operation), selection valve (valve position) and potentiostat (potential applied, data acquisition); each step was represented by a line in the sia.vi programme in figure 2. After defining the steps, a *step sequence* was created. A step sequence consisted of the proper succession of steps that constituted the desired experimental procedure as illustrated in the sia.vi programme in figure 2. Steps could be inserted in the sequence in any order and the sequence was terminated by typing ‘0’ as the last step. A coloured indicator lit up to highlight the current step in the sequence. When a step with the scan control ‘enabled’ was encountered in the step sequence, the voltammetric programme sw was automatically initiated by the main programme sia.vi.

The programme sw allowed selection of the SW parameters to be used for the voltammetric scan (frequency, scan increment, pulse height and time window for current acquisition during each pulse) and was a modification of a programme developed earlier [15]. When invoked by the main programme, sw digitally created the SW potential–time signal, performed the voltammetric scan, acquired the current in proper synchronization with the applied potential and, finally, displayed the voltammogram.

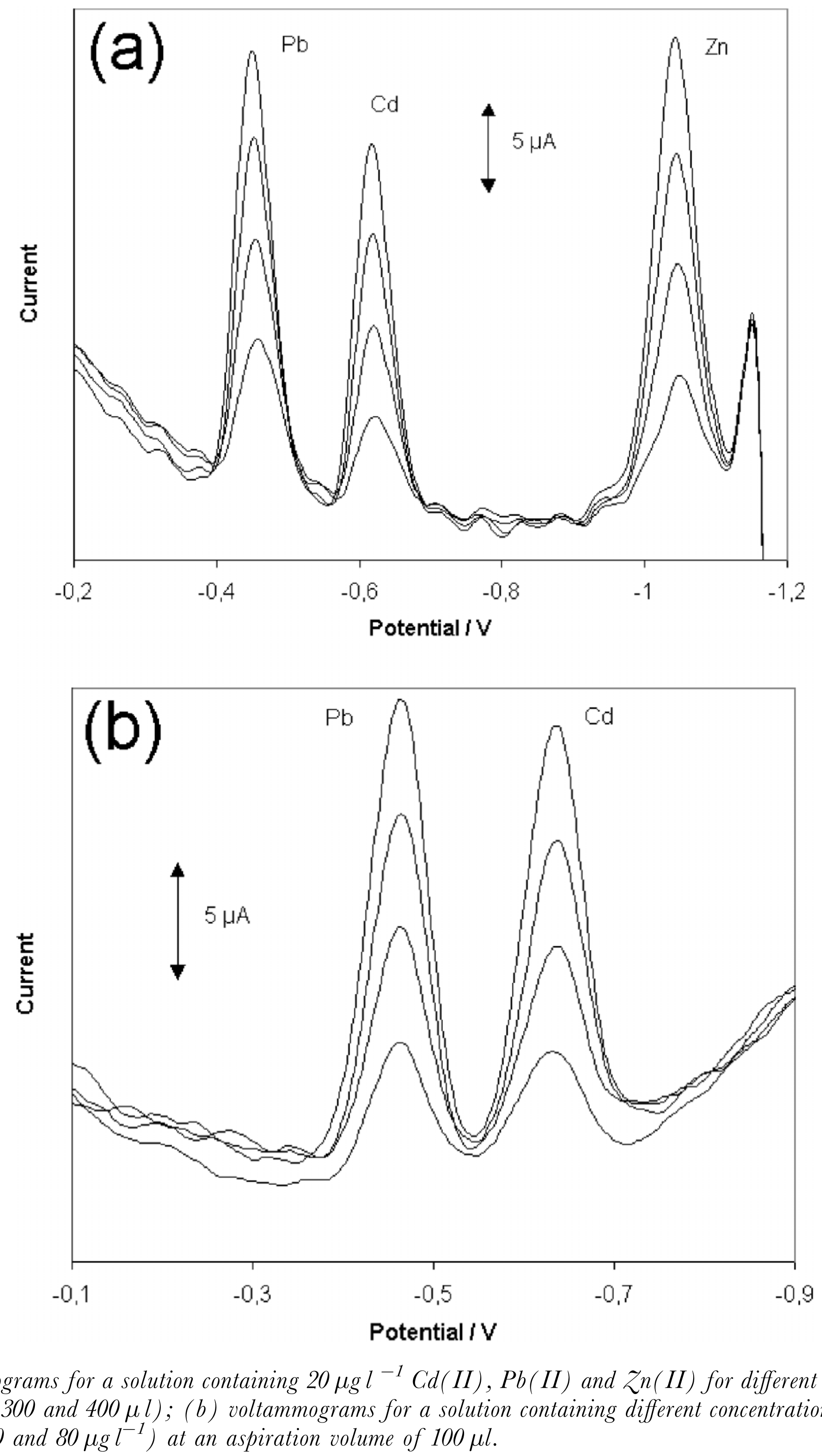

*Figure 3. (a) Voltammograms for a solution containing 20 µg l$^{-1}$ Cd(II), Pb(II) and Zn(II) for different sample aspiration volumes (from bottom: 100, 200, 300 and 400 µl); (b) voltammograms for a solution containing different concentrations of Cd(II) and Pb(II) (from bottom: 20, 40, 60 and 80 µg l$^{-1}$) at an aspiration volume of 100 µl.*

### Results and discussion

The main advantage of SIA as applied to stripping analysis is that all the instrumental experimental conditions can be conveniently controlled and varied readily and rapidly. The instrumental experimental variables include the deposition time, the sample (and ligand) volumes, the deposition potential, the mass-transfer conditions and the SW parameters. The sensitivity in stripping analysis will be strongly dependent on the proper selection of all these parameters.

### *Anodic stripping voltammetry*

For the SWASV experiments, Cu(II), Pb(II), Cd(II) and Zn(II) were selected as test analytes. These metals were preconcentrated on the MFE by electrolytic accumulation followed by an anodic stripping scan:

$$M^{2+} + 2e^- \rightarrow M(Hg) \quad \text{(preconcentration)}$$

$$M(Hg) \rightarrow M^{2-} + 2e^- \quad \text{(stripping),}$$

where $M^{n+}$ are the metal ions and M(Hg) is the metal–mercury amalgam.

*Table 1. Experimental conditions for the determination of different species by square-wave stripping analysis.*

| Species | Ni(II), Co(II)[a] | U(VI)[a] | Cu(II), Cd(II), Pb(II), Zn(II)[b] |
|---|---|---|---|
| Technique | SWAdSV | SWAdSV | SWASV |
| Ligand | 0.05 mmol $l^{-1}$ DMG in carrier | 0.1 mol $l^{-1}$ cupferron in carrier | – |
| Preconcentration potential (V) | −0.7 | 0 | −1.2 or −1 |
| Cleaning potential (V) | 1.3 | −0.7 | 0.6 |
| Potential scan | −0.7 to −1.3 | 0 to −0.7 | −1.2 to 0.2 |
| Carrier | ammonia buffer, pH 9 | acetate buffer, pH 4.5 | acetate buffer, pH 4.5 |
| Frequency (Hz) | 25 | 25 | 50 |
| Pulse height (mV) | 40 | 40 | 20 |
| Step increment (mV) | 2 | 2 | 4 |
| Hg(II) solution | 200 mg $l^{-1}$ in 0.1 mol $l^{-1}$ HCl | 200 mg $l^{-1}$ in 0.1 mol $l^{-1}$ HCl | 10 mg $l^{-1}$ in 0.1 mol $l^{-1}$ HCl |

[a]Preplating, [b]*in-situ* plating.

*Table 2. Step sequence for the determination of metals by square-wave adsorptive stripping voltammetry with preplated mercury-film electrodes.*

| Step | Duration (s) | Pump speed (ml $min^{-1}$) | Pump status | Valve position | Electrode potential (V) | Description |
|---|---|---|---|---|---|---|
| 1[a] | 30 | 1.2 | deliver | 7 | +0.2 | deliver carried to fill main flow line |
| 2[a] | 10 | 1.2 | aspirate | 1 | +0.2 | aspirate Hg(II) solution into holding coil |
| 3[a] | 20 | 1.2 | deliver | 2 | +0.2 | flush holding coil |
| 4[b] | 10 | 1.2 | aspirate | 3 | +0.2 | aspirate sample into holding coil |
| 5[b] | 20 | 1.2 | deliver | 2 | +0.2 | flush holding coil |
| 6 | 10 | 1.2 | aspirate | 1 | +0.2 | aspirate Hg(II) solution into holding coil |
| 7 | 10 | 1.2 | aspirate | 3 | +0.2 | sample aspiration into holding coil |
| 8 | 100 | 0.6 | deliver | 7 | preconcentration potential | deliver sample and Hg(II) to electrode for preconcentration |
| 9 | 10 | 0 | deliver | 7 | preconcentration potential | equilibration |
| 10 | variable | 0 | deliver | 7 | potential scan | stripping and recording of voltammogram |
| 11 | 5 | 1.8 | deliver | 7 | +0.6 | deliver carrier for electrode cleaning |

The main analysis cycle is enclosed in a frame.
[a]Required at start-up; [b]required at start-up and when changing the sample.

The experimental conditions of the determinations are shown in table 1 and the sequence of operations is shown in table 2.

Initially, the main flow line (i.e. the tubing from the holding coil to the waste) and the flow lines connecting the valve to the Hg(II) solution and the sample were filled with the corresponding solutions (steps 1–5). These steps were only required at start-up and when the sample was changed.

The main analysis sequence was then initiated (steps 6–11). The sample solution and the Hg(II) solution were aspirated into the holding coil (step 10). The zones were delivered to the cell for electrolytic *in-situ* deposition of the metals and mercury on the electrode at the selected preconcentration potential (step 12). In step 9, the solution was left to equilibrate and the voltammetric scan was initiated from the initial to the final potential (step 10). Note that the stripping step was carried out in the carrier solution and not in the sample solution, enabling the automated implementation of the so-called 'medium exchange' approach. Finally, the electrode was cleaned from the remaining analytes and the mercury film in the flowing carrier (step 11). The next preconcentration/stripping cycle started from step 6.

In the case of ASV, the preconcentration efficiency, and therefore the sensitivity, could be controlled by varying the volume of the sample aspirated and/or the residence time of the sample in the cell. The effect of varying the volume of the sample is shown in figure 3(a), suggesting that the sensitivity increased in proportion to the volume of the aspirated sample. Figure 3(b) shows voltammograms for the simultaneous determination of Cd and Pb in increasing concentrations in steps of 20 $\mu g\, l^{-1}$.

### *Adsorptive stripping voltammetry*

For the SWAdSV experiments, Ni(II), Co(II) and U(VI) were selected as test analytes. These metals were preconcentrated on the MFE by adsorptive accumula-

*Table 3. Step sequence for the determination of metals by square-wave adsorptive stripping voltammetry with preplated mercury-film electrodes.*

| Step | Duration (s) | Pump speed ($ml\,min^{-1}$) | Pump status | Valve position | Electrode potential (V) | Description |
|---|---|---|---|---|---|---|
| 1[a] | 30 | 1.2 | deliver | 7 | +0.2 | deliver carried to fill main flow line |
| 2[a] | 10 | 1.2 | aspirate | 1 | +0.2 | aspirate Hg(II) solution into holding coil |
| 3[a] | 20 | 1.2 | deliver | 2 | +0.2 | flush holding coil |
| 4[a] | 10 | 1.2 | aspirate | 5 | +0.2 | aspirate $KNO_3$ solution |
| 5[a] | 20 | 1.2 | deliver | 2 | +0.2 | flush holding coil |
| 6[b] | 10 | 1.2 | aspirate | 3 | +0.2 | aspirate sample into holding coil |
| 7[b] | 20 | 1.2 | deliver | 2 | +0.2 | flush holding coil |
| 8[c,d] | 10 | 1.2 | aspirate | 5 | +0.2 | aspirate $KNO_3$ solution |
| 9[c] | 20 | 1.2 | aspirate | 1 | +0.2 | aspirate Hg(II) solution into holding coil |
| 10[c,d] | 10 | 1.2 | aspirate | 5 | +0.2 | aspirate $KNO_3$ solution |
| 11[c] | 120 | 0.6 | deliver | 7 | −1 | deliver Hg(II) solution to electrode for Hg plating |
| 12 | 10 | 1.2 | aspirate | 3 | −1 | sample aspiration into holding coil |
| 13 | 10 | | aspirate | 4 | −1 | ligand aspiration into holding coil |
| 14 | 100 | 0.6 | deliver | 7 | preconcentration potential | deliver sample and ligand to electrode for on-line complexation and accumulation |
| 15 | 10 | 0 | deliver | 7 | preconcentration potential | equilibration |
| 16 | variable | 0 | deliver | 7 | potential scan | stripping and recording of voltammogram |
| 17 | 10 | 0.6 | deliver | 7 | cleaning potential | deliver carrier for Hg cleaning |
| 18[c] | 15 | 1.8 | deliver | 7 | +0.6 | deliver carrier for Hg stripping |

The main analysis cycle is enclosed in a frame.
[a]Required at start up; [b]required at start up and when changing the sample; [c]required when forming and changing the mercury-film; [d]required only for alkaline carriers.

tion of their complexes with a surface-active ligand (dimethylglyoxime for Ni(II) and Co(II) and cupferron for U(VI)), followed by a cathodic scan:

$$Mn^{+} + nL^{-} \rightarrow MLn_{sol} \quad \text{(complexation in solution)}$$

$$MLn_{sol} \rightarrow MLn_{ads} \quad \text{(absorption on the electrode)}$$

$$MLn_{ads} + ne^{-} \rightarrow M + nL^{-} \quad \text{(stripping),}$$

where $L^{-}$ is the complexing agent and $MLn_{sol}$ is the adsorbed complex on the electrode surface.

The experimental conditions of the determinations are shown in table 1 and the sequence of operations in table 3. Initially, the main flow line (i.e. the tubing from the holding coil to the waste) and all the flow lines connecting the valve to the Hg(II) solution, the $KNO_3$ solution and the sample were filled with the corresponding solutions (steps 1–7). These steps were only required at start-up and when the sample was changed.

The mercury film was then plated by aspirating a zone of the mercury plating solution. In some cases, it was necessary to isolate the mercury plating solution from the carrier solution. This was required when the carrier was alkaline to avoid the hydrolysis of Hg(II) ions. This was accomplished by inserting the zone of the mercury plating solution between two zones of $0.1\,mol\,l^{-1}$ $KNO_3$ solution that served as buffers between the mercury plating solution and the carrier. The zones of the $0.1\,mol\,l^{-1}$ $KNO_3$, the mercury plating solution and the $0.1\,mol\,l^{-1}$ $KNO_3$ were sequentially aspirated into the holding coil (steps 8–10, respectively), followed by delivery of the Hg(II) solution to the cell for mercury plating (step 11). This procedure was only required for the formation of a new film.

The main analysis sequence was then initiated (steps 12–17). The sample solution was aspirated into the holding coil (step 12). The solution containing the ligand was aspirated adjacent to the sample in the holding coil (step 13). The zone(s) was delivered to the cell for adsorptive deposition of the analyte(s) on the MFE (step 14). In step 15, the solution was left to equilibrate and the voltammetric scan was initiated from the initial to the final potential (step 16). As in the case of ASV, stripping was performed in the carrier solution, allowing the application of 'medium exchange'. Finally, the electrode was cleaned from the remaining analytes in the flowing carrier (step 17). The next preconcentration/stripping cycle started from step 12.

An additional step (18) was employed for stripping off the mercury film after a series of measurements. In this case, the next mercury plating/preconcentration/stripping cycle started from step 8.

Figure 4(a) shows a voltammogram for the determination of Ni(II) and Co(II). In the case of metal ions accumulated as their complexes with different ligands, the complex formation efficiency could be controlled by altering the zone penetration between the ligand and sample zone; preconcentration will take place only from the fraction of volume in which the two zones overlap. Using a constant sample volume and decreasing the delivery flow rate caused a modest increase in the stripping peak heights because the effective residence

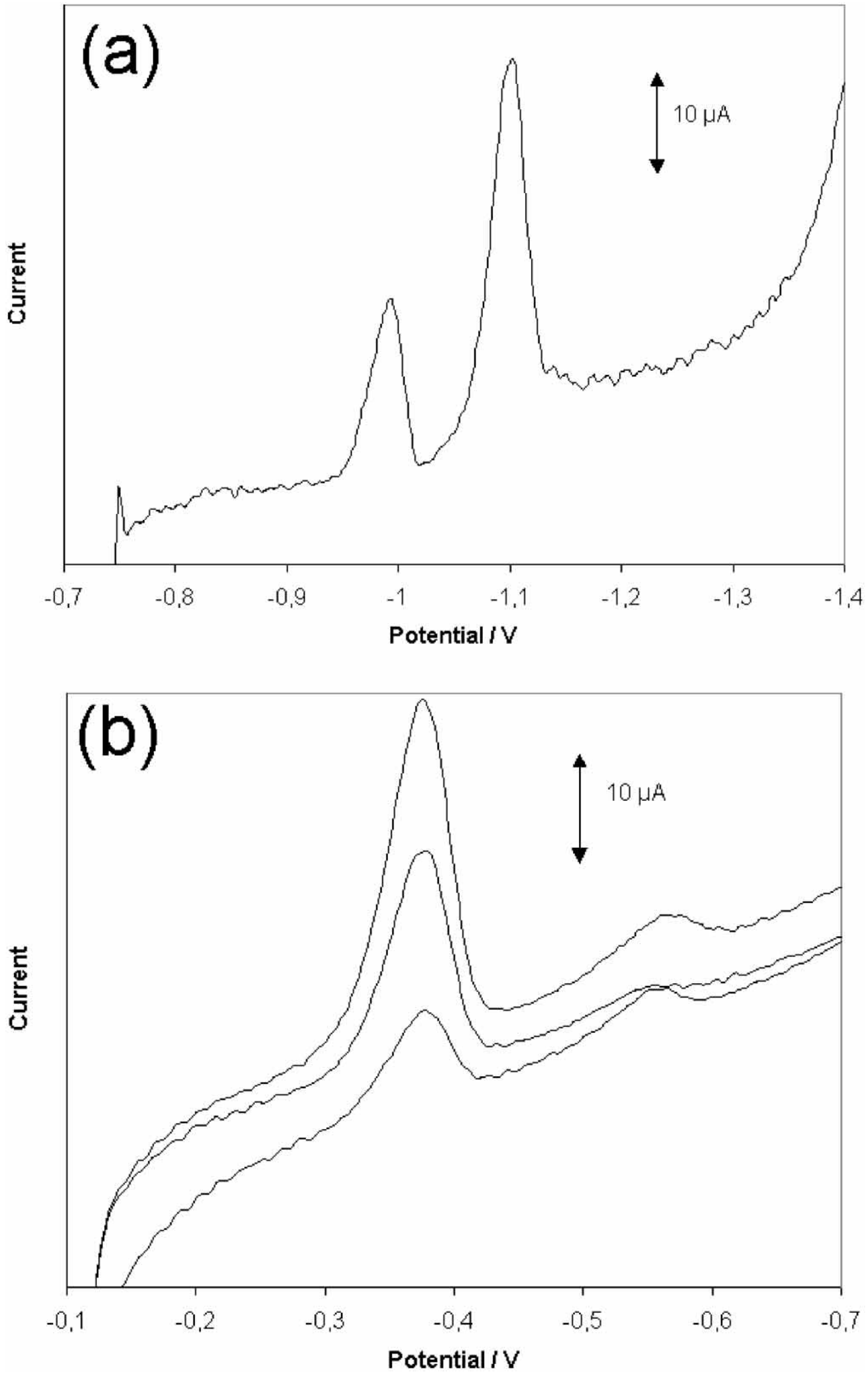

*Figure 4. (a) Voltammogram for a solution containing 20 μg $l^{-1}$ Co(II) and Ni(II) for a sample aspiration volume of 200 μl and a ligand aspiration volume of 200 μl; (b) voltammogram for a solution containing 20 μg $l^{-1}$ U(VI) using the multiple-zone approach for a sample aspiration volume of 200 μl and a ligand aspiration volume of 200 μl (from bottom: one, two and four repeats).*

time of the overlapped zones in the cell was increased and also zone penetration between the sample and the ligand zones was enhanced (due to an increase in dispersion). However, to further increase the sensitivity, another procedure was used. Instead of aspirating a single sample zone and a single ligand zone, multiple zones of sample and ligand were alternatively aspirated in the holding coil. In the context of the step sequence in table 3, this procedure was easily accomplished by repeating steps 12 and 13. Figure 4(a) shows the utility of the multiple zone approach in the sensitivity for U(VI) determination.

### *Application*

The SIA system developed was applied to the determination of Cu(II) and Pb(II) in a phosphate fertilizer. In this case, 2.0 g fertilizer was dissolved in 10 ml concentrated $HClO_4$ and the solution was diluted to 100 ml. The solution was allowed to settle and a sample

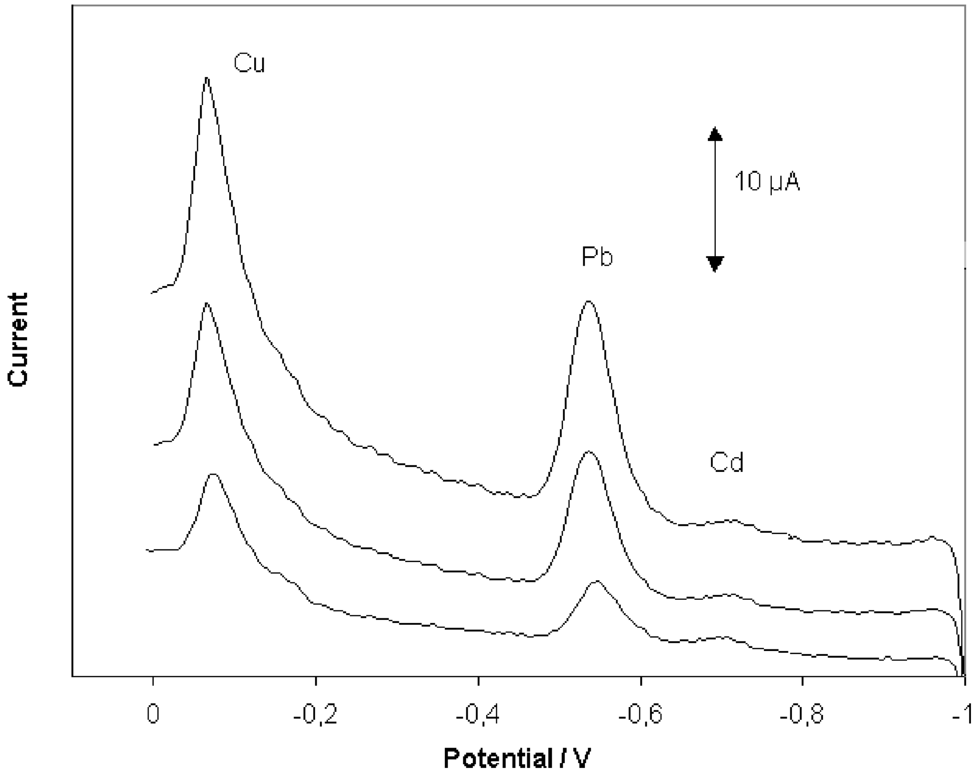

*Figure 5. Determination of Pb(II) and Cu(II) in a phosphate fertiliser sample using an aspiration volume of 200 µl (from bottom: sample, sample + 20 µg l$^{-1}$ Cu(II) + 20 µg l$^{-1}$ Pb(II), and sample + 60 µg l$^{-1}$ Cu(II) + 30 µg l$^{-1}$ Pb(II)).*

was obtained from the clear supernatant solution. The sample was injected into the SIA manifold and the voltammogram was recorded. Injection of the sample was repeated twice after standard additions of Cu(II) and Pb(II) and the corresponding voltammograms were recorded; the three voltammograms are shown in figure 5. The concentration of Cu(II) and Pb(II) in the sample was calculated using the peak heights in the standard addition calibration curve. The content calculated was 0.9 mg kg$^{-1}$ Pb and 2.5 mg kg$^{-1}$ Cu in the sample. These values were in good statistical agreement with the results from atomic absorption spectrometry used as a reference method. In this application, the preconcentration potential was set to −1.0 V to avoid the co-deposition of Zn (that forms an intermetallic compound with Cu on the MFE surface and interferes with the analysis). The peak of Cd in this sample was discernible but not measurable under the experimental conditions used in this work.

### References

1. Lenehan, C. E., Barnett, N. W. and Lewis, S. W., *Analyst*, **127** (2002), 997.
2. Ivaska, A. and Kubiak, W. W., *Talanta*, **44** (1997), 713.
3. Kubiak, W. W., Latonen, R. M. and Ivaska, A., *Talanta*, **53** (2001), 1211.
4. Economou, A. and Fielden, P. R., *Analyst*, **128** (2003), 205.
5. Economou, A. and Fielden, P. R., *Trends in Analytical Chemistry*, **16** (1995), 286.
6. Osteryoung, J. and O'Dea, J. J., in Bard, A. J. (Ed.), *Electronalytical Chemistry*, 14 (New York: Marcel Dekker), 209–308.
7. Suteerapataranon, S., Jakmunee, J., Vaneesorn, Y. and Grudpan, K., *Talanta*, **58** (2002), 1235.
8. Armalis, S. and Kubiliene, E., *Analytica Chimica Acta*, **423** (2000), 287.
9. Economou, A. and Fielden, P. R., *Talanta*, **46** (1998), 1137.
10. Greenway, G. M. and Wolfbauer, G., *Analytica Chimica Acta*, **312** (1995), 15.
11. Economou, A., Volikakis, G. J. and Efstathiou, C. E., *Journal of Automated Methods and Management in Chemistry*, **21** (1999), 33.
12. Allerhand, A. and Dobie-Galuska, A., *Chemical Educator*, **5** (2000), 71.
13. Fletcher, P. J. and Van Staden, J. F., *Analytica Chimica Acta*, **499** (2003), 123.
14. Rocha, I. and Ferreira, E. C., *Analytica Chimica Acta*, **462** (2002), 293.
15. Economou, A., Bolis, S. D., Efstathiou, C. E. and Volikakis, G. J., *Analytica Chimica Acta*, **467** (2002), 179.

